# Genome-wide identification and expression analysis of the HD2 protein family and its response to drought and salt stress in *Gossypium* species

**DOI:** 10.3389/fpls.2023.1109031

**Published:** 2023-02-13

**Authors:** Nasreen Bano, Shafquat Fakhrah, Rayees Ahmad Lone, Chandra Sekhar Mohanty, Sumit Kumar Bag

**Affiliations:** ^1^ Council of Scientific & Industrial Research (CSIR)-National Botanical Research Institute (CSIR-NBRI), Lucknow, India; ^2^ Academy of Scientific and Innovative Research (AcSIR), Ghaziabad, India; ^3^ Department of Botany, University of Lucknow, Lucknow, India

**Keywords:** expression, phylogenetic analysis, conserved, co-expression, pathways, gene ontology

## Abstract

Histone deacetylase 2 (HD2) proteins play an important role in the regulation of gene expression. This helps with the growth and development of plants and also plays a crucial role in responses to biotic and abiotic stress es. HD2s comprise a C_2_H_2_-type Zn^2+^ finger at their C-terminal and an HD2 label, deacetylation and phosphorylation sites, and NLS motifs at their N-terminal. In this study, a total of 27 HD2 members were identified, using Hidden Markov model profiles, in two diploid cotton genomes (*Gossypium raimondii* and *Gossypium arboretum*) and two tetraploid cotton genomes (*Gossypium hirsutum* and *Gossypium barbadense*). These cotton HD2 members were classified into 10 major phylogenetic groups (I-X), of which group III was found to be the largest with 13 cotton HD2 members. An evolutionary investigation showed that the expansion of HD2 members primarily occurred as a result of segmental duplication in paralogous gene pairs. Further qRT-PCR validation of nine putative genes using RNA-Seq data suggested that GhHDT3D.2 exhibits significantly higher levels of expression at 12h, 24h, 48h, and 72h of exposure to both drought and salt stress conditions compared to a control measure at 0h. Furthermore, gene ontology, pathways, and co-expression network study of GhHDT3D.2 gene affirmed their significance in drought and salt stress responses.

## Introduction

Plants are immobile organisms and encounter a variety of stresses throughout the course of their lives. To overcome these stressful conditions, plants usually adopt three mechanisms (Larcher et al., 1973). First, they avoid stress through temporal activity. Second, they develop greater resistance against stressors by increasing their tolerance, and reduce their sensitivity through greater plasticity. Third, they have recovery mechanisms that can be stimulated in case of damage, e.g., reconstruction of damaged tissues (Huey et al., 2002). Post-translational modifications (PTMs) in plants play a significant role in combating environmental stresses (Mazzucotelli et al., 2008; Hashiguchi and Komatsu, 2016; De Vega et al., 2018). These processes are governed by certain proteins and enzymes. Histone deacetylases (HDACs) are a group of proteins involved in post-translational histone modification (histone deacetylation). This group is essential in controlling gene expression, which in turn modifies biological processes by removing acetyl moieties from histone proteins, resulting in the restoration of a positive charge (Dangl et al., 2001; Han et al., 2016).

There are three classes of HDACs: silent information regulator 2 (SIR2), reduced potassium dependency 3 (RPD3) or histone deacetylase 1 (HDA1), and histone deacetylase 2 (HD2) (Pandey et al., 2002) (Taunton et al., 1996; Wu et al., 2000; Tian and Chen, 2001; Pandey et al., 2002; Liu et al., 2014; Zhao et al., 2014; Han et al., 2016). The histone deacetylase 2 (HD2 or type 2 HDACs) subfamily of HDACs is recognized as an essential unit in controlling the structure and function of ribosomal chromatin, development, immunity, ribosomal RNA processing, hormonal signaling, and environmental stresses in plants (Lusser et al., 1997; Ding et al., 2012; Han et al., 2016). To date, HD2 members have only been reported to be found in plants, and not in animals or fungi (Pandey et al., 2002; Ma et al., 2013). HD2 has been isolated from maize embryos as an acidic nucleolar phosphoprotein (Lusser et al., 1997). HD2A, HD2B, HD2C, and HD2D are four HD2 members that have been identified in *Arabidopsis thaliana* (Wu et al., 2000; Dangl et al., 2001; Pandey et al., 2002; Zhou et al., 2004); these are also known as HDT1, HDT2, HDT3, and HDT4. Computational analysis of the HD2 domain showed highly conserved motif sequences. In monocots as well as in dicots, nucleotide sequence analysis has demonstrated that a single HD2 gene directs the entire set of HD2 members during development (Pandey et al., 2002; Han et al., 2016).

HD2 proteins can repress gene expression *via* modification of histones, as demonstrated by the finding that methylation and deacetylation of H3 Lys9 are mediated *via* AtHD2A (Lawrence et al., 2004). All HD2s are located in the nucleus (Lusser et al., 1997; Lawrence et al., 2004; Zhou et al., 2004; Han et al., 2016), and global HD2 expression has been observed in *A. thaliana* (Lawrence et al., 2004; Zhou et al., 2004; Hollender and Liu, 2008). AtHD2A and AtHD2B have been demonstrated to be involved in determining the polarity of leaves in *A. thaliana* (Ueno et al., 2007), while the AtHD2C gene plays a crucial role in abiotic stress responses and has a significant function in abscisic acid biosynthesis (Sridha and Wu, 2006). The AtHD2D gene also contributes to plant development and abiotic stress responses (Han et al., 2016). HD2s have been thoroughly studied in dicots as well as in monocots, such as *Nicotiana tabacum* (Nicolas-Frances et al., 2018), *Solanum chacoense* (Demetriou et al., 2009), *Dimocarpus longan* (Kuang et al., 2012; Grandperret et al., 2014), *Zea mays* (Dangl et al., 2001; Pandey et al., 2002; Demetriou et al., 2009), *Oryza sativa* (Fu et al., 2007; Ding et al., 2012), and *Hordeum vulgare* (Demetriou et al., 2009; Grandperret et al., 2014).

Globally, the most important crop for the production of natural textile fabric is cotton (*Gossypium* spp.). *Gossypium* is a good model for the study of the evolution, origins, and domestication of polyploid species (Hu et al., 2019). It contains 5 tetraploid and 45 diploid species (Li et al., 2014; Chen et al., 2020). Due to its broader adaptability, the cultivated species *G. hirsutum* produces high-yield cotton fiber with moderate fiber quality; this species alone is responsible for approximately 90% of annual world cotton output (Hu et al., 2019).

It has been shown that the identified HD2 genes play a crucial role in growth, development, and responses to biotic and abiotic stresses in plants. Previous investigation of the histone deacetylase (HDAC) gene family in diploid and allotetraploid cotton (Imran et al., 2020) has primarily emphasized the different fiber development stages (0-25 DPA). In the present study, we mainly focus on drought and salt stress conditions at different time scales.

Over the last several years, whole genome sequencing of cotton (*G. hirsutum, G. barbadense, G. arboreum*, and *G. raimondii*) has been completed (Wang et al., 2012a; Li et al., 2014; Liu et al., 2015b). The completion of genome sequencing has enabled the identification of HD2 members in these four cotton genomes, as well as enabling evolutionary, expression, and co-expression studies. In *G. hirsutum*, the patterns of expression of putative HD2 members under drought and salt stress conditions have also been examined. The present study aimed to establish a foundation for an understanding of the evolutionary history of these genes, their functional importance, and their significance in drought and salt stress responses. This study was expected to provide considerable novel findings on epigenetic regulation, representing insight into its functional and evolutionary roles in *Gossypium* species.

## Methodology

### Sequence retrieval and genome-wide identification of the HD2 gene family in *Gossypium* species

The whole-genome protein sequence dataset for *G. arboreum*, *G. barbaense*, and *G. hirsutum* was downloaded from the CottonGen database (https://www.cottongen.org/), and the dataset for *G. raimondii* was obtained from Phytozome, version 12 (https://phytozome.jgi.doe.gov/pz/portal.html) (Nordberg et al., 2014). Total protein sequences for other plant species from different taxonomic groups were downloaded from the website of the National Center for Biotechnology Information (NCBI) (http://www.ncbi.nlm.nih.gov/). A total of 58 HD2 protein sequences from 42 plant species were obtained from the NCBI and utilized to construct Hidden Markov model (HMM) profiles. This profile of the HD2 domains (the HD2 label and the catalytic, regulatory, and zinc- finger domains) was employed as a query to identify HD2 gene family members using HMMER (V3.0) (Finn et al., 2015). Protein sequences and CDSs for *G. arboreum, G. raimondii, G. hirsutum*, and *G. barnadense* were also downloaded from the CottonGen database (https://www.cottongen.org/) (Yu et al., 2014). All hits were queried in the Pfam (http://pfam.xfam.org/) and InterProScan (http://www.ebi.ac.uk/interpro/search/sequence-search/) databases to verify the presence of conserved domains. The ProtParam (http://web.expasy.org/protparam/) tool offered by Expasy was used to estimate the physicochemical parameters of *Gossypium* HD2 proteins. The ProtParam tool was also used to estimate biophysical and biochemical properties, such as number of amino acids, molecular weight, grand average hydropathy (GRAVY), theoretical isoelectric point (pI), aliphatic index, and instability index. The cotton HD2 gene subfamilies were named as per the orthologous HD2 members in the *A. thaliana* genome.

### Subcellular localization, HD2 protein domain structure, and nuclear localization signal prediction

The CELLO server, v.2.5 (http://cello.life.nctu.edu.tw/), was used to predict the probable subcellular locations of all the cotton HD2 proteins identified. The Pfam database was used to analyze the protein domain structures, and the Illustrator for Biological Sequences software package (http://ibs.biocuckoo.org/) (Liu et al., 2015a) was subsequently utilized to construct a schematic diagram of protein functional domains. Nuclear localization signals were examined using Motif Scan (https://myhits.isb-sib.ch/cgi-bin/motif_scan).

### Multiple sequence alignment, classification, and phylogenetic tree construction for HD2 members

HD2 protein sequences were extracted from the complete genome sequences of 39 plant species. Multiple sequence alignments (MSAs) of full-length HD2 protein sequences with identified cotton HD2 proteins were performed using the Clustal X program (http://www.clustal.org/) (Larkin et al., 2007) with default parameters. Subsequently, these aligned sequences were used to construct a phylogenetic tree. The MEGA 7 software package (http://www.megasoftware.net/) (Kumar et al., 2016) was utilized to build an unrooted phylogenetic tree using the maximum likelihood (ML) method with the following parameters: pairwise gap deletion, JTT matrix-based model, and 1000 bootstrap values (Felsenstein, 1985).

### Chromosomal mapping and gene duplication analysis

The physical (genomic) locations on chromosomes of all *Gossypium* HD2 genes were obtained by cross-referencing the results of searching for HD32 CDS sequences using the BLASTN search program against the resources of the CottonGen cotton database (https://www.cottongen.org/) and the Phytozome database, version 12 (https://phytozome.jgi.doe.gov/pz/portal.html). All HD2 genes of *Gossypium* species were mapped on the chromosomes using the MapInspect software (http://mapinspect.software.informer.com/).

Paralogous HD2 genes were identified through reciprocal BLAST analysis with e-value <10^-5^ in order to deduce the evolutionary mechanism of the HD2 gene family in *Gossypium* species (*G. arboreum*, *G. raimondii*, *G. hirsutum*, and *G. barbadense*). As per the reciprocal BLAST output, duplication events in *Gossypium* HD2 genes were determined using the MCScanX toolkit (Wang et al., 2012b). Additionally, Ka/Ks analysis of ortholog and paralog sequences was carried out using the PAL2NAL program. Subsequently, Ks values were used to compute the approximate dates of duplication and speciation events *via* the formula T = Ks/2λ, where λ is the assumed value of the clock-like rate. In addition, the Ka/Ks ratio was used as an indicator of selection pressure for the duplicated HD2 genes. Specifically, Ka/Ks ratios greater than 1, less than 1, and equal to 1 were taken to suggest positive, negative, and neutral (purifying selection) evolution, respectively (Zhang et al., 2006).

### Gene structure and conserved motif analysis

In *G. arboreum*, *G. raimondii*, *G. hirsutum*, and *G. barbadense* species, the gene structures of identified HD2 genes were examined *via* comparison of the predicted HD2 coding sequences with their corresponding genomic sequences using the GSDS online tool (Gene Structure Display Server; http://gsds.cbi.pku.edu.cn/) (Hu et al., 2015).

A MEME (Multiple Expectation maximization for Motif Elicitation) tool (http://meme.nbcr.net/meme/) (Bailey et al., 2009) was used for the identification of conserved protein motifs in identified cotton HD2 proteins. The following parameters were used in this analysis: optimum width = 6 to 300; one per sequence; maximum number of motifs to find = 20. Furthermore, these motifs were annotated using the InterProScan program (Quevillon et al., 2005).

### Patterns of expression of cotton HD2 family members under drought and salt stress

To investigate the expression pattern of cotton HD2 members under salt and drought stress conditions, raw Illumina RNA-Seq data for *G. hirsutum* (accession number: PRJNA532694) were obtained from the Sequence Read Archive of the National Center for Biotechnology Information (the NCBI-SRA database). The raw reads were filtered using the Trimmomatic tool (Bolger et al., 2014), and high-quality reads were subsequently mapped on the *G. hirsutum* genome (Wang et al., 2019) using the STAR aligner (Dobin et al., 2013) with default parameters. Transcript abundance was estimated using the StringTie software (Pertea et al., 2015), and the differential expression of genes was evaluated using the edgeR Bioconductor package (Robinson et al., 2010).

### RNA isolation, cDNA synthesis, and quantitative real-time PCR validation of HD2 family members in *G. hirsutum*

The regulatory mRNA sequences of HD2 family members were validated *via* qRT-PCR. Stress conditions were employed on eight-week-old *G. hirsutum* plants. The plants were raised in pots (30 cm diameter, 25 cm height, 12 liter capacity) at the CSIR-NBRI garden, with soil made up of 40% silt, 20% clay, and 40% sand. They were exposed to a minimum temperature of 27°C and a maximum of 34°C; there were 14 hours of light and 10 hours of darkness per day. To create conditions of drought and salt stress, plants (in triplicate) were administered a single treatment of 20% PEG8000 solution (Shafiq et al., 2015) and 300-mM NaCl (Wei et al., 2017). Untreated plants were taken as controls. During the experiment, leaves were taken from the topmost juvenile section of each plant at the 0h, 12h, 24h, 48h, and 72 h time points after administration of the stress treatment. RNA was extracted from these samples using a Spectrum™ Plant Total RNA Isolation kit (Sigma, USA). cDNA was prepared with 1µg RNA using the Verso cDNA synthesis kit (Thermo Scientific) following the provided protocol. 2× diluted cDNA was utilized to examine the expression of nine selected putative genes *via* a fluorescent quantitative detection system using qRT-PCR (HiMedia Insta Q48 M4); a 20 µl reaction was prepared with 10 µl SYBER™ green master mix (Applied Biosystems), along with 5 pmol of primer concentration. Primers were designed with the help of Primer-BLAST, and ubiquitin was taken as a normalizing control. The qRT-PCR program was set to apply a temperature of 95°C for 2 min, followed by 40 cycles of denaturation at 95°C for 15 s and annealing at 60°C for 1 min. Gene expression levels were calculated with reference to ubiquitin for normalization, and the relative expression levels of the selected genes at each time point were compared with their expression levels at 0h. CT values were calculated with mean ± SD by the2-∆CT method (Livak and Schmittgen, 2001).

### Co-expression network, metabolic pathway, and gene ontology analyses of genes positively and negatively co-expressed with GhHDT3D.2

Based on the higher expression of GhHDT3D.2 observed in RNA-Seq and in qRT-PCR values, the co-expression network of GhHDT3D.2 was constructed for both stress conditions (drought and salt). This co-expression network was built using FPKM values using the “expression correlation networks” module of Cytoscape version 3.8.2 (Otasek et al., 2019). Specifically, this module was used to identify positive and negative Pearson correlations (r ≥ 0.95 and r ≤ - 0.95) between interacting members of the network. Co-expressed genes and networks were visualized using Cytoscape in a circular force-directed layout. The key metabolic pathways of genes positively and negatively co-expressed with GhHDT3D.2 (PCoEGs and NCoEGs) were evaluated using the PageMan software, version 3.5.1 (Thimm et al., 2004). To determine their functional categories, the average statistical test Benjamini Hochberg test were employed. Gene ontology (GO) analysis for three categories (biological processes, molecular functions, and cellular components) was carried out using agriGO, v2.0 (http://systemsbiology.cau.edu.cn/agriGOv2/). Singular enrichment analysis (SEA), with statistical testing at a p-value threshold of < 0.05, was used to retrieve GO annotations.

### *Cis*-regulatory element analysis

To analyze the *cis*-regulatory elements in the upstream sequences of HD2 genes, 1.5 kb upstream region sequences of 27 cotton HD2 genes were obtained from the CottonGen (https://www.cottongen.org/) and Phytozome v12 (https://phytozome.jgi.doe.gov/pz/portal.html) databases. *Cis*-regulatory elements were identified using the PlantCARE database (http://bioinformatics.psb.ugent.be/webtools/plantcare/html/) (Lescot et al., 2002).

## Results

### Identification of HD2 members in *Gossypium* species

Four cotton species, viz., *G. arboreum, G. raimondii, G. hirsutum*, and *G. barbadense*, were used to identify HD2 members. HD2 domain sequences were obtained from the NCBI and used to construct Hidden Markov model (HMM) profiles. The HMMER search program was used to identify HD2 orthologs by querying against these four *Gossypium* species. The HD2 gene was later analyzed for similarity and conserved domains by comparing data from the Pfam and InterproScan databases. A total of 27 HD2s were identified (**Table 1**): 4 GaHD2s (*G. arboreum*), 5 GrHD2s (*G. raimondii*), 9 GbHD2s (*G. barbadense*), and 9 GhHD2s (*G. hirsutum*). Number of amino acids, instability index, grand average hydropathy (GRAVY), theoretical isoelectric point (pI), molecular weight, and aliphatic index are important physiological parameters in determining the primary structure of proteins. The properties of the identified cotton HD2s, including protein length (aa), gene name, locus ID, isoelectric point (pI), molecular weight (Da), chromosome location, number of introns, sub-cellular localization, aliphatic index, instability index, and GRAVY, were analyzed using the ProtParam tool. Additionally, the subcellular localizations of predicted HD2 members were identified using the CELLO v2.5. There was wide variation in the length of cotton HD2 proteins, ranging from 201 to 299 amino acids in *G. arboreum*, 272 to 304 amino acids in *G. raimondii*, 240 to 304 amino acids in *G. hirsutum*, and 285 to 376 amino acids in *G. barbadense.* Isoelectric points ranged from 4.52 to 6.02, 4.19 to 5.06, 4.50 to 4.86, and 4.86 to 5.29 in *G. arboreum, G. raimondii, G. barbadense*, and *G. hirsutum*, respectively. Polypeptide molecular weight ranged from 21.588 kDa to 32.484 kDa in *G. arboreum*, 29.604 kDa to 32.838 kDa in *G. raimondii*, 26.563 kDa to 32.875 kDa in *G. hirsutum*, and 30.424 kDa to 40.694 kDa in *G. barbadense.* Interestingly, *Gossypium* HD2s contained a large number of introns; this count varied from 5 to 8, 7 to 9, 5 to 9, and 7 to 11 in *G. arboreum, G. raimondii, G. hirsutum*, and *G. barbadense*, respectively. Cotton HD2 members were found to be located in the nucleus (**Table 1**).

**Table 1:** HD2 genes in *G. arboreum, G. raimondii, G. hirsutum*, and *G. barbadense*, and their properties.

Gene Name	Gene ID	Chromosome location^a^	Length (aa)	MW (Da)	pI	No. of introns	Subcellular localization
GaHDT1.2	Cotton_A_38986	CA_chr11:13342769:13344503:+	281	29702.47	4.89	7	Nuclear
GaHDT1.1	Cotton_A_13908	CA_chr10:70332895:70334925:+	237	25020.70	6.02	8	Nuclear
GaHDT3.1	Cotton_A_00517	CA_chr2:68281323:68284053:+	299	32484.44	4.84	7	Nuclear
GaHDT3.2	Cotton_A_05503	CA_chr4:129649767:129652788:+	201	21588.11	4.52	5	Nuclear
GbHDT3A.1	Gbar_A01G020670.1	Gbar_A01:113313343:113316535:+	302	32849.83	4.88	7	Nuclear
GbHDT3A.2	Gbar_A11G034200.1	Gbar_A11:112495331:112498709:-	302	32765.54	4.75	7	Nuclear
GbHDT3D.2	Gbar_D01G021820.1	Gbar_D01:60890069:60893373:+	303	32748.59	4.85	7	Nuclear
GbHDT3D.1	Gbar_D11G036570.1	Gbar_Scaffold3329:569711:573101:+	302	32745.58	4.70	7	Nuclear
GbHDT1A.1	Gbar_A09G010350.1	Gbar_A09:58078435:58081066:-	285	30424.82	4.74	9	Nuclear
GbHDT2A	Gbar_A07G019940.1	Gbar_A07:83611088:83616185:-	374	40694.83	4.50	11	Nuclear
GbHDT1A.2	Gbar_A05G008180.1	Gbar_A05:7511022:7513548:-	295	31441.89	4.63	9	Nuclear
GbHDT1D.1	Gbar_D09G010030.1	Gbar_D09:34681744:34684364:-	286	30467.98	4.86	9	Nuclear
GbHDT1D.2	Gbar_D05G008680.1	Gbar_D05:7072085:7074613:-	306	32605.27	4.67	9	Nuclear
GhHDT3A.2	Ghir_A11G034800.1	Ghir_A11:122526638:122531536:-	302	32738.51	4.75	8	Nuclear
GhHDT3D.1	Ghir_D01G021720.1	Ghir_D01:61035852:61038556:+	247	26565.20	5.29	7	Nuclear
GhHDT3D.2	Ghir_D11G035640.1	Ghir_D11:72330837:72334110:-	304	32875.68	4.70	7	Nuclear
GhHDT1A.2	Ghir_A09G010110.1	Ghir_A09:61537456:61540486:-	279	29734.24	4.91	5	Nuclear
GhHDT2A	Ghir_A07G020200.1	Ghir_A07:88450751:88452795:-	240	26563.23	5.04	7	Nuclear
GhHDT1A.1	Ghir_A05G008720.1	Ghir_A05:7996063:7998807:-	295	31441.89	4.63	9	Nuclear
GhHDT1D.2	Ghir_D09G009840.1	Ghir_D09:36139490:36142155:-	286	30433.96	4.86	9	Nuclear
GhHDT1D.1	Ghir_D05G008730.1	Ghir_D05:7126741:7129344:-	293	31377.94	4.64	9	Nuclear
GhHDT3A.1	Ghir_A01G020190.1	Ghir_A01:115297731:115300457+	270	29486.23	4.96	8	Nuclear
GrHDT3.2	Gorai.007G371300.1	Chr07:60425967:60429311:-	304	32838.54	4.70	7	Nuclear
GrHDT3.1	Gorai.002G243000.2	Chr02:60721336:60724290:+	272	29604.58	5.06	7	Nuclear
GrHDT4	Gorai.002G242800.1	Chr02:60691443:60699441:+	287	31537.94	4.19	7	Nuclear
GrHDT1.2	Gorai.006G109300.1	Chr06:35338435:35341077:-	286	30392.91	4.85	9	Nuclear
GrHDT1.1	Gorai.009G088600.1	Chr09:6468177:6470775:-	301	32102.71	4.76	8	Nuclear

^a^Chromosomal location: ‘+’ and ‘-’ indicate the forward and reverse strand, respectively.

Proteins with an instability index < 40 are considered stable, whereas those with a value > 40 are considered unstable. The volume occupied by aliphatic side chains relative to the overall volume of the protein is termed the “aliphatic index”. In *G. arboreum, G. raimondii, G. hirsutum*, and *G. barbadense*, instability index values for HD2 genes ranged from 42.44 to 63.31, 37.93 to 62.94, 35.89 to 62.35, and 42.13 to 62.35, respectively; and aliphatic index values varied from 39.35 to 56.65, 45.48 to 60.62, 44.75 to 57.75, and 44.41 to 65.80, respectively. Thus, the majority of the predicted HD2 members were hydrophilic in nature and unstable, a finding consistent with the instability index measure. However, some HD2s (such as GhHDT3D.1, GhHDT3A.1, and GrHDT3.1) were stable. Moreover, HD2 proteins had negative GRAVY scores, indicating their hydrophilic behavior; however, the degree of hydrophilicity demonstrated a greater variability (**Table 2**).

**Table 2:** Protein properties of different HD2s.

Cotton HD2s	Instability index	Aliphatic index	Grand average of hydropathicity (GRAVY)	Protein stability
GaHDT3.1	42.44	49.60	-1.093	unstable
GaHDT1.1	60.23	51.18	-0.862	unstable
GaHDT1.2	49.15	56.65	-0.904	unstable
GaHDT3.2	63.31	39.35	-1.273	unstable
GbHDT1D.1	52.56	44.41	-1.074	unstable
GbHDT1A.2	62.35	44.75	-1.093	unstable
GbHDT1A.1	52.08	44.88	-1.099	unstable
GbHDT1D.2	56.86	47.94	-1.042	unstable
GbHDT3A.2	49.62	49.11	-1.046	unstable
GbHDT3D.1	49.71	48.48	-1.011	unstable
GbHDT3D.2	45.72	49.27	-1.118	unstable
GbHDT3A.1	42.13	50.07	-1.083	unstable
GbHDT2A	56.55	65.80	-0.741	unstable
GhHDT1D.2	52.56	45.77	-1.071	unstable
GhHDT1A.1	62.35	44.75	-1.093	unstable
GhHDT1A.2	50.23	45.84	-1.057	unstable
GhHDT3A.2	50.90	49.11	-1.037	unstable
GhHDT3D.2	48.95	47.83	-1.010	unstable
GhHDT1D.1	55.74	49.73	-1.071	unstable
GhHDT3D.1	35.89	53.81	-0.939	stable
GhHDT3A.1	38.97	52.04	-1.003	stable
GhHDT2A	51.66	57.75	-0.970	unstable
GrHDT1.2	50.60	45.77	-1.062	unstable
GrHDT1.1	62.94	45.48	-1.104	unstable
GrHDT3.2	50.00	47.83	-1.039	unstable
GrHDT3.1	37.93	60.62	-0.842	stable
GrHDT4	44.89	58.08	-1.055	unstable

### Domain structure analysis of cotton HD2s

**Figure 1:**
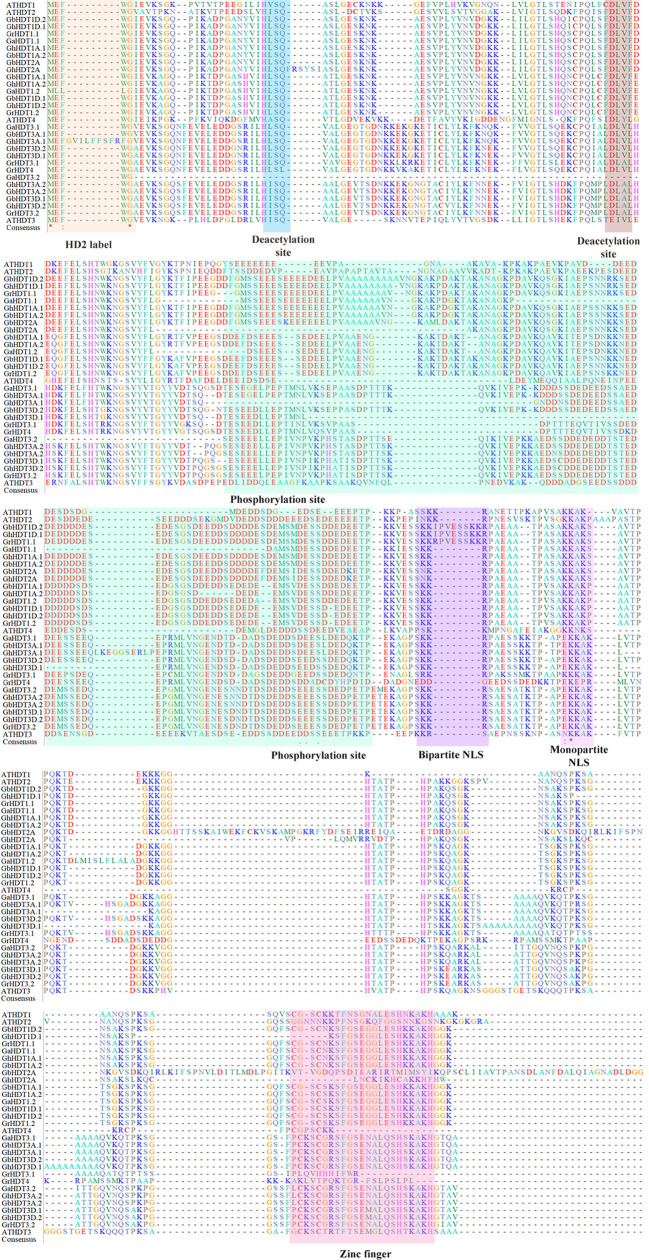
Results of multiple sequence alignment (MSA) of cotton HD2 proteins with Arabidopsis HD2s. MSA of 27 putative *Gossypium* HD2 proteins and four Arabidopsis HD2 proteins performed by Clustal X. Orange, blue, brown, violet, and pink- colored shading indicates the conserved sites (HD2 label, phosphorylation sites, bipartite NLS, monopartite NLS, and zinc finger, respectively) of a typical HD2 protein. Green, yellow, and blue letters with an asterisk represent amino acid conservation in all the domains.

**Figure 2:**
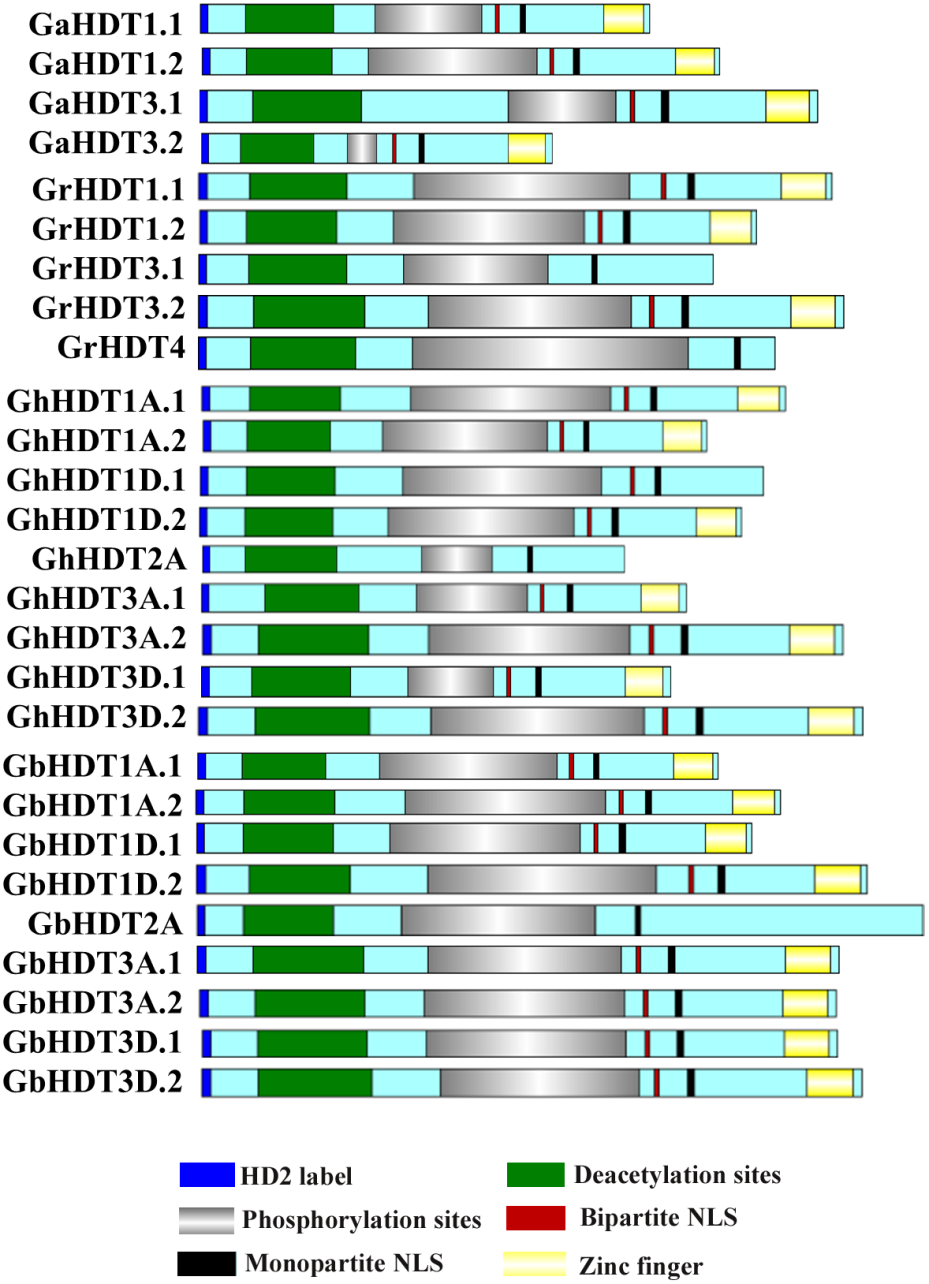
Schematic depiction of GaHD2s, GrHD2s, GhHD2s, and GbHD2s. The domain structures of GaHD2s, GrHD2s, GhHD2s, and GbHD2s, drawn using Illustrator for Biological Sequences.

**Figure 3:**
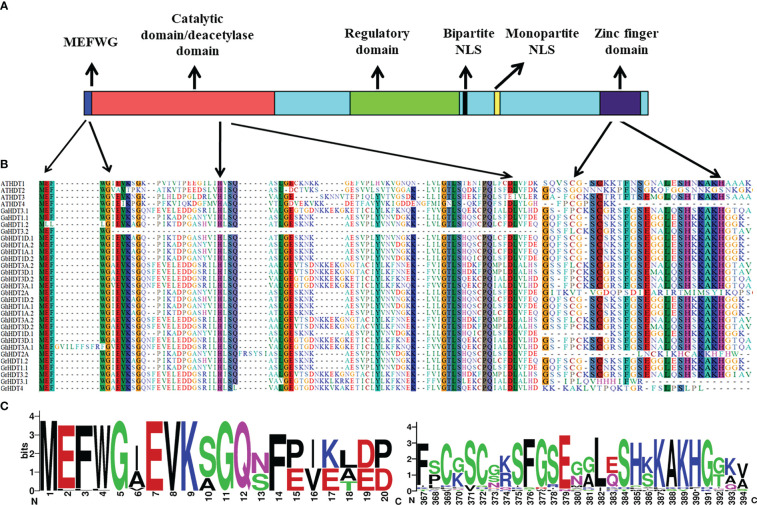
Conservation of HD2 domains in all cotton HD2 proteins. **(A)** Functionally confirmed motifs of *Arabidopsis* HD2s. **(B)** Alignment and sequence logo of the conserved MEFWG pentapeptide sequence of 27 cotton HD2s with that of 4 AtHD2s. **(C)** Alignment and sequence logo of the conserved zinc finger domain of 27 cotton HD2s with that of 4 AtHD2s.

In order to perform multiple sequence alignment (MSA) of cotton HD2 protein sequences, ClustalX 2.1 was used. Comparison of cotton HD2s *via* the Pfam and InterPro protein domain databases revealed that they contained highly conserved domains relative to the structure of a typical HD2 protein (**Figure 1**). These results indicated that GaHDT1.1, GaHDT1.2, GaHDT3.1, and GaHDT3.2 contained the HD2 label, a deacetyl/catalytic domain, phosphorylation sites, mono- and bipartite NLS motifs, and a zinc finger domain from N-terminus to C-terminus. Similarly, GrHDT1.1, GrHDT 1.2, GrHDT 3.2, GhHDT1A.1, GhHDT1A.2, GhHDT1D.2, GhHDT3A.1, GhHDT3A.2, GhHDT3D.1, GhHDT3D.2, GbHDT1A.1, GbHDT1A.2, GbHDT1D.1, GbHDT1D.2, GbHDT3A.1, GbHDT3A.2, GbHDT3D.1, and GbHDT3D.2 contained all the conserved domains. In contrast, GrHDT3.1, GrHDT4, GhHDT2A, and GbHDT2A did not contain a bipartite NLS motif or zinc finger domain, and GhHDT1D.1 did not contain a zinc finger domain at the C-terminus end (**Figures 2**, **3**). A monopartite NLS motif was predicted to be present in all the cotton HD2 proteins, suggesting that this might generate a signal that drives cotton HD2s to the nucleus. Most HD2 proteins were between 232 and 384 amino acids in length. A highly correlated relationship exists between amino acid sequence variation and the length of the highly variable regulatory domain, which resides in the center (Bourque et al., 2016). These results suggest a high degree of homology between the amino acids in these conserved cotton HD2s and AtHD2s (**Figure 3**).

### Multiple sequence alignment and evolutionary analysis

**Figure 4:**
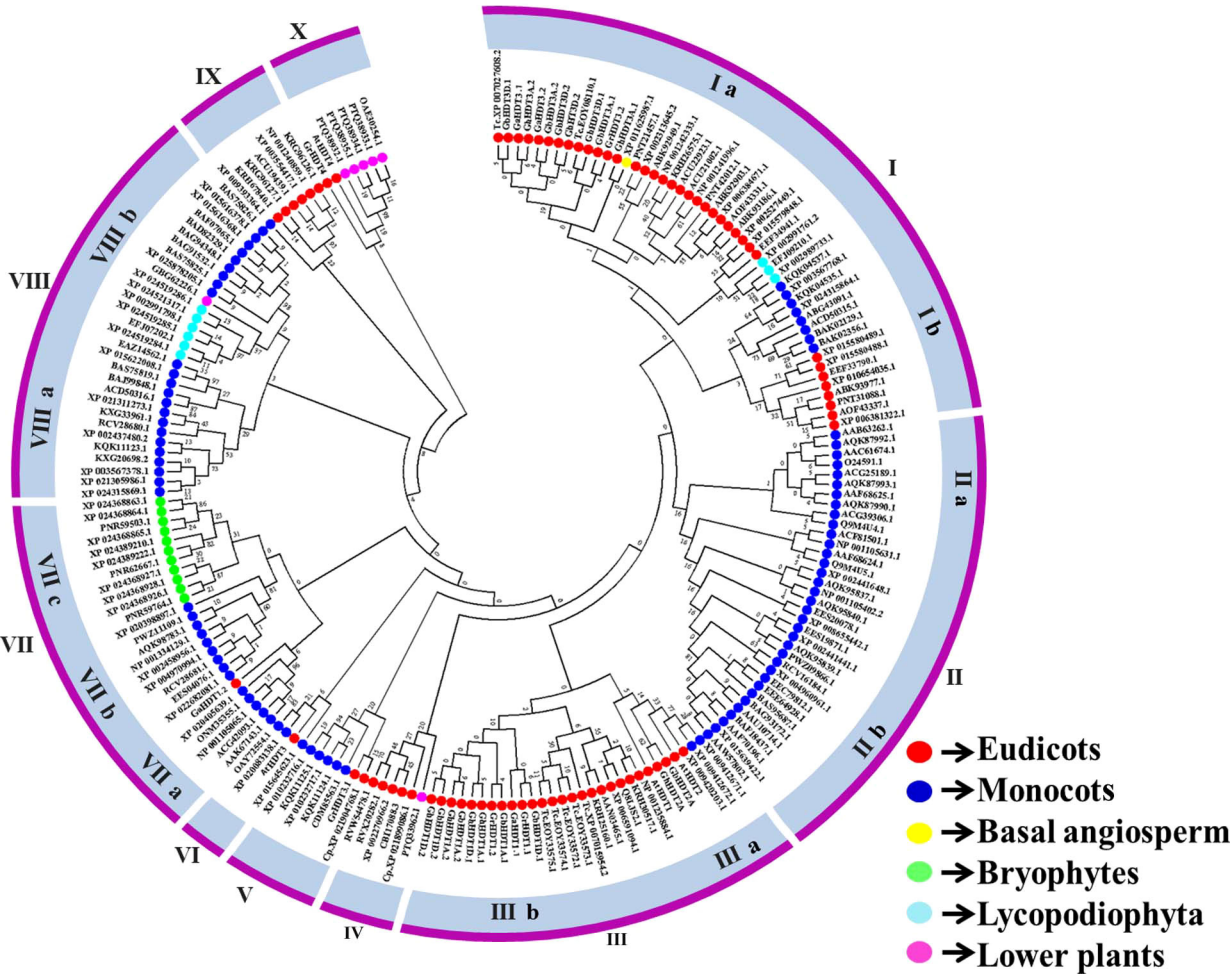
Phylogenetic relationships of cotton HD2s with other plant species and *Arabidopsis thaliana*.

To evaluate the classification of HD2 genes in cotton, the sequence features of 44 plant species were analyzed, and an unrooted phylogenetic tree of the HD2 genes was constructed (**Figure 4**). Subsequently, an investigation of the evolutionary relationships between HD2 proteins in cotton and those in other plant species was carried out. For this purpose, MSA was conducted on 27 cotton HD2 proteins and 198 HD2 proteins from other plant species (basal angiosperm, bryophytes, lower plants, Lycopodiophyta, monocots, and eudicots) (**Table 3**). The maximum likelihood (ML) method was used for phylogenetic tree construction. The nomenclature of cotton HD2s was done as per *A. thaliana* HD2s.

**Table 3:** List of plants entered into phylogenetic analysis and number of HD2 genes identified in 44 sequenced plants, animalia, and fungi.

Lineage	Family	Species	Common name	Genome size	No. of HD2 genes
Algae	Chlorellaceae	*Chlorella vulgaris*	Chlorella	~37.34 Mbp	0
	Chlamydomonadaceae	*Chlamydomonas reinhardtii*	Single-cell green alga	~120 Mbp	0
	Volvocaceae	*Volvox carteri*	Colonial green algae	~125 Mbp	0
Animalia	Drosophilidae	*Drosophila Melanogaster*	Common fruit fly	~122.65 Mbp	0
	Rhabditidae	*Caenorhabditis elegans*	Free-living transparent nematode	~100.25 Mbp	0
	Muridae	*Mus musculus*	House mouse	~2.6 Gbp	0
	Hominidae	*Homo sapiens*	Human	~3.67 Gbp	0
Basal angiosperm	Amborellaceae	*Amborella trichopoda*	Amborella	~706.33 Mbp	1
Bryophytes	Funariaceae	*Physcomitrella patens*	Spreading earthmoss	~480 Mbp	11
Fungi	Blastocladiaceae	*Allomyces macrogynus*	Allomyces	~52.62 Mbp	0
	Undefined	*Batrachochytrium dendrobatidis*	Amphibian chytrid fungus	~24.13 Mbp	0
	Ancylistaceae	*Conidiobolus coronatus*	Entomophthora coronata	~31.71 Mbp	0
	Unikaryonidae	*Encephalitozoon cuniculi*	–	~2.8 Mb	0
	Sordariaceae	*Neurospora crassa*	Red bread mold	~43 Mbp	0
	Schizophyllaceae	*Schizophyllum commune*	Split-gill mushroom	~37.93 Mbp	0
Lower plants	Salviniaceae	*Azolla filiculoides*	**-**	~0.75 Gbp	0
	Characeae	*Chara braunii*	–	~1.71 Gbp	1
	Cycadaceae	*Cycas revoluta*	Kungi (comb) palm	~9.08 Gbp	0
	Funariaceae	*Funaria hygrometrica*	Common cord-moss	–	0
	Marchantiaceae	*Marchantia polymorpha*	Umbrella liverwort	~210.56 Mbp	6
	Pinaceae	*Pinus contorta*	Shore pine	~21.82 Gbp	0
	Zygnemataceae	*Spirogyra maxima*	–	–	0
Lycopodiophyta	Selaginellaceae	*Selaginella moellendorffii*	Spikemoss	~212.46 Mbp	9
Monocots	Musaceae	*Musa acuminata*	Dwarf banana	~600 Mbp	4
	Poaceae	*Brachypodium distachyon*	Purple false brome	~270 Mbp	11
		*Hordeum vulgare*	Barley	~5.3 Gbp	5
		*Oryza sativa*	Rice	~500 Mbp	22
		*Setaria italica*	Foxtail millet	~490 Mbp	6
		*Sorghum bicolor*	Great millet	~730 Mbp	11
		*Triticum aestivum*	Bread wheat	~17 Gbp	2
		*Zea mays*	Maize	~2.4 Gbp	29
Eudicots	Brassicaseae	*Arabipsis thaliana*	Mouse-ear cress	~135 Mbp	4
	Malvaceae	*Theobroma cacoa*	Cacao tree	~445 Mbp	7
		*Gossypium arborium*	Cotton	~1746 Mbp	4
		*Gossypium barbadense*	Cotton	~2631 Mbp	9
		*Gossypium hirsutum*	Cotton	~2400 Mbp	9
		*Gossypium raimondii*	Cotton	~880 Mbp	5
	Caricaceae	*Carica papaya*	Papaya	~372 Mbp	2
	Euphorbiaceae	*Ricinus communis*	Castor bean	~320 Mbp	6
	Fabaceae	*Glycine max*	Soybean	~1115 Mbp	17
	Salicaceae	*Populus trichocarpa*	Populus	~500 Mbp	12
	Vitaceae	*Vitis vinifera*	Grape	~500 Mbp	5
Gymnosperm	Pinaceae	*Pinus taeda*	Loblolly pine	~20.1 Gbp	0
	Ginkgoaceae	*Ginkgo biloba*	Maidenhair tree	~9.765 Gbp	0

Based on the phylogenetic relationships plant HD2 genes were clustered into ten major groups (I to X), and cotton HD2 proteins were classified into four of these groups, with none belonging to Group II, IV, VI, VIII, or IX. Groups I, II, III, VII, and VIII were further divided into two, two, two, three, and two subgroups respectively (labeled a, b, and c). Group III contained the largest number of cotton HD2 genes (13 members) followed by Group I with 11 and Groups V, VIII, and IX, with one HD2 member each (**Figure 4**).

### Phylogenetic tree, gene structure, and protein motif analyses of *Gossypium* HD2 genes

**Figure 5:**
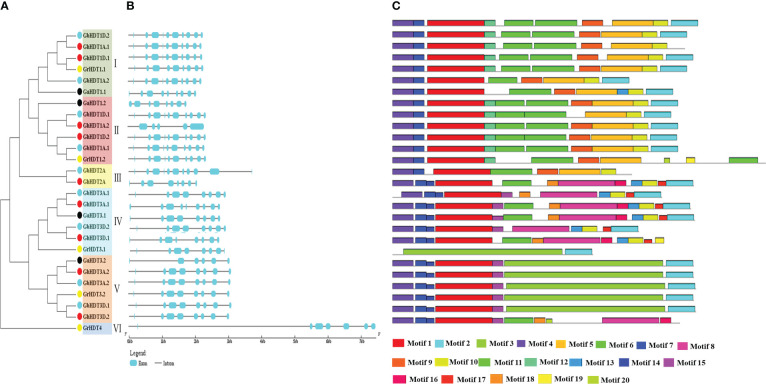
Phylogenetic tree, conserved protein motifs, and gene structure analysis of HD2 members found in *G. arboreum, G. raimondii, G. barbadense, and G. hirsutum*. **(A)** Phylogenetic tree of *G. arboreum, G. raimondii, G. hirsutum, and G. barbadense* constructed *via* the ML method using 1000 bootstrap values. Different dot colors represent different *Gossypium* species (black: *G. arborium*; yellow: *G. raimondii*; red: *G. hirsutum*; sky blue: *G. barbadense*. Sub-families I, II, III, IV, V, and VI are shown in green, red, yellow, sky blue, and orange, respectively. **(B)** Representation of the gene structure of cotton HD2 members. Cyan boxes and black lines represent exons and introns, respectively. **(C)** Conserved protein motifs of HD2s, as determined using the MEME tool. Each motif is denoted by a specific color.

To understand the evolutionary relationships and gain insight into the structural diversity and similarity of *Gossypium* HD2s, a separate maximum likelihood tree was constructed with 1000 bootstrap values using the HD2 protein sequences of *Gossypium* species. The topology of the tree, HD2 duplicating nodes, conserved motifs, and exon/intron distribution enabled the categorization of the cotton HD2s into six sub-families (I - VI, shown in different background colors in **Figure 5**). Genes within the same sub-family with the highest levels of identity (>80%), indicate divergent evolution from a common ancestor, in the orthologous gene pairs (**Figure 5A**).

To understand the structural variations in cotton HD2s, coding sequences and the corresponding genome sequences were compared to determine their exon/intron arrangements. Interestingly, the majority of cotton HD2 gene structures within the same clade were similar, i.e., group-specific exon/intron patterns were observed. The majority of cotton HD2 members had introns ranging from 5 to 1. Specifically, the number of introns varied from 5 to 8 (GaHD2s), 7 to 9 (GrHD2s), 5 to 9 (GhHD2s), or 7 to 11 (GbHD2s) (**Figure 5B**). The gene structure of all cotton HD2s was analyzed and compared in order to examine the stability of the exon/intron members with respect to the structure of the phylogenetic tree. It was found that the majority of cotton HD2s falling under the same clade shared similar arrangements of exons/introns. For example, in subfamily I, the HD2 gene comprised 9 introns with 10 CDS (with the exceptions of GrHDT1.1 and GaHDT1.1), while members of the V group comprised 7 introns with 8 CDS (with the exceptions of GaHDT3.2, GhHDT3A.2, and GrHDT4, which consisted of 5, 8, and 7 introns with 0, 8, and 7 CDS and 6, 0, and 0 exons, respectively).

A motif-based sequence analysis tool (MEME) was utilized to examine the conserved motifs in HD2 proteins. The InterProScan was used to annotate these motifs. A total of 20 conserved motifs were determined in the *Gossypium* HD2 members. Motifs 1 and 4 were found in the database and annotated as nucleoplasmin-like domains. These have been reported to be important players in governing the dynamic architecture of the chromatin (Singh et al., 2022) (**Figure 5C** and **Supplementary Table 1**). Motif 4 (HD2 pentapeptide, shown in violet) was approximately present in all the cotton HD2s and showed a conserved N-terminus pentapeptide sequence. Motif 2, annotated as a zinc finger domain, was found in all the cotton HD2 proteins except GhHDT1D.1, GbHDT2A, GhHDT2A, GrHDT3.1, and GrHDT4 (**Figure 5C**). Additionally, motifs 3 and 5 were annotated as histone deacetylase- like domains and were found to be present in all cotton HD2 proteins except GaHDT1.2, GbHDT2A, GhHDT3A.1, and GhHDT3D.1 members. Most of the cotton HD2s exhibited a similar motif composition to their close evolutionary relatives; therefore, it is speculated that they might have similar functions (**Figure 5C**).

### Chromosomal distribution and duplication events in *Gossypium* HD2 genes

**Figure 6:**
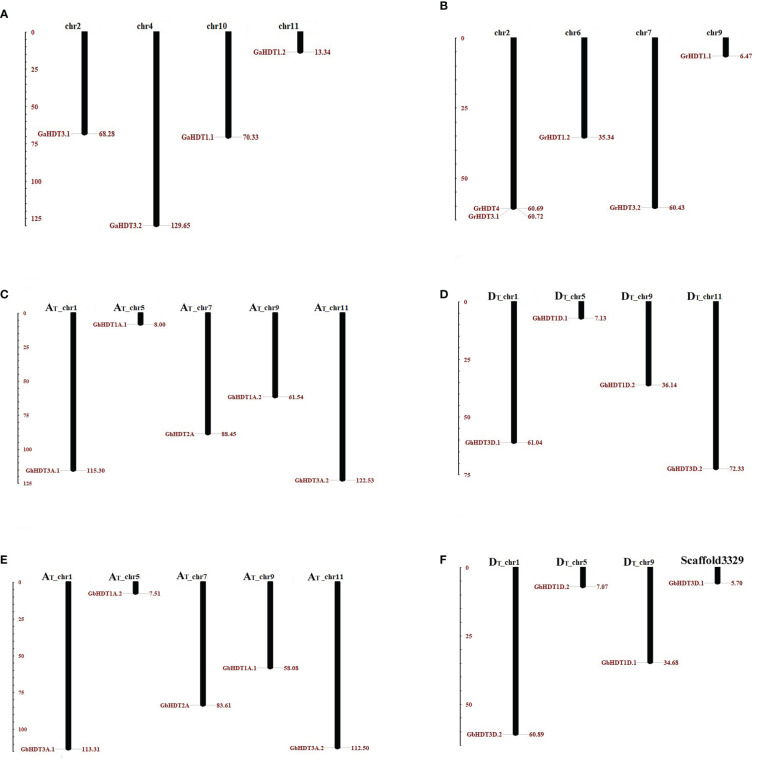
Chromosomal localization of HD2 family genes in the *Gossypium* genome. In this physical mapping, the positions of HD2 members on A, D, and AD genomes are illustrated separately. HD2 genes are indicated by red-brown lines on *Gossypium* chromosomes, and the number of the chromosome depicted is provided at the top. **(A, B)** The distribution of HD2 genes in the *G. arboreum* and *G. raimondii* genomes. **(C, D)** The arrangement of HD2 genes in the *G. hirsutum* genome; **(E, F)** The distribution HD2 genes in the *G. barbadense* genome. The scale is measured in mega bases (Mb).

In order to determine the chromosomal distribution within the HD2 gene family in *Gossypium*, BLASTN search was carried out for all GaHD2, GrHD2, GhHD2, and GbHD2 members in *G. arboreum, G. raimondii, G. barbadense*, and *G. hirsutum*. After all the *Gossypium* genes had been mapped to their corresponding chromosomes, GaHD2 genes were localized on chromosomes 2, 4, 10, and 11 (**Figure 6A**), while the distribution of GrHD2 genes consisted of locations on chromosomes 2, 6, 7, and 9 (**Figure 6B**). Similarly, in *G. hirsutum*, more HD2 genes were positioned on A_T_ (1, 5, 7, 9, 11) than on D_T_ chromosomes (1, 5, 9, 11), with 5 and 4 genes, respectively (**Figures 6C, D**); in *G. barbadense*, a larger number of HD2 genes were also located on A_T_ (1, 5, 7, 9, 11) than on D_T_ chromosomes (1, 5, 9, scaffold3329), with 5 and 4 genes, respectively (**Figures 6E, F**).

**Figure 7:**
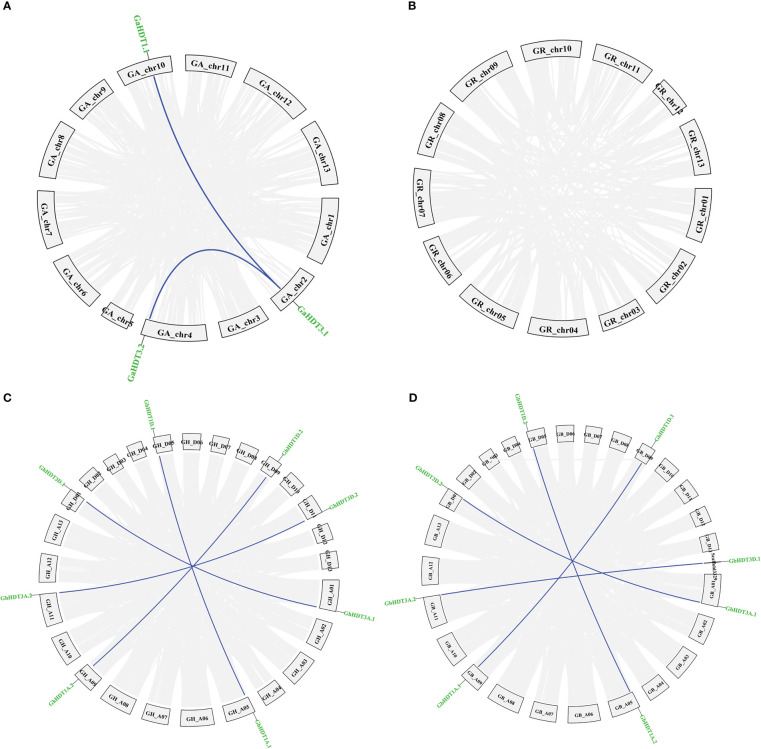
Paralogous duplicated HD2 gene pairs in *Gossypium* genomes (*G. arboreum, (G) raimondii, (G) barbadense*, and *G. hirsutum)*. Depiction of duplicated genes in **(A)** *G. arborium*, **(B)** *G. raimondii*, **(C)** *G. hirsutum*, and **(D)** *G. barbadense*. Duplicated HD2 genes are indicated by dark blue lines; collinear blocks are indicated by grey lines.

Analysis of gene duplication events identified two pairs of paralogous HD2 members in *G. arboreum* (**Figure 7A**), while no paralogous genes were detected in *G. raimondii* (**Figure 7B**). Furthermore, four pairs of paralogous HD2 members were identified in *G. hirsutum* and *G. barbadense* (**Figures 7C, D**). As shown in **Figure 7**, all paralogous gene pairs were found to be located on distinct chromosomes, providing a considerably strong indication that *Gossypium* HD2 members were expanded through segmental duplication. In *G. arborium*, two segmental duplications (GaHDT1.1/GaHDT3.1 and GaHDT3.1/GaHDT3.2) occurred between 45.53 and 18.06 MYA. Furthermore, in *G. hirsutum*, four segmental duplications (GhHDT3A.1/GhHDT3D.1, GhHDT1A.1/GhHDT1D.1, GhHDT1A.2/GhHDT1D.2, and GhHDT3A.2/GhHDT3D.2) occurred 2.51, 1.32, 2.12, and 1.42 MYA; finally, in *G. barbadense*, four segmental duplications (GbHDT3A.1/GbHDT3D.2, GbHDT1A.2/GbHDT1D.2, GbHDT1A.1/GbHDT1D.1, and GbHDT3A.2/GbHDT3D.1) were detected before 1.76, 1.44, 2.09, and 1.60 MYA (**Table 4**).

**Table 4:** Ka/Ks ratios and dates of duplication for duplicated HD2 genes in *G. arboreum, G. hirsutum, and G. barbadense*.

Name of duplicated HD2 gene 1	ID of duplicated HD2 gene 1	ID of duplicated HD2 gene 2	Name of duplicated HD2 gene 2	Ka	Ks	Ka/Ks	Date (MYA); T = Ks/2λ	Selective pressure	Duplicate type
GaHDT1.1	Cotton_A_00517	Cotton_A_00517	GaHDT3.1	0.346280551	1.3663338	0.253437741	45.54445998	Purifying selection	Segmental
GaHDT3.1	Cotton_A_05503	Cotton_A_05503	GaHDT3.2	0.145578276	0.54183266	0.268677557	18.06108875	Purifying selection	Segmental
GbHDT3A.1	Gbar_D01G021820.1	Gbar_D01G021820.1	GbHDT3D.2	0.024334196	0.05283875	0.460536965	1.761291524	Purifying selection	Segmental
GbHDT1A.2	Gbar_D05G008680.1	Gbar_D05G008680.1	GbHDT1D.2	0.007225489	0.04341175	0.166440851	1.44705849	Purifying selection	Segmental
GbHDT1A.1	Gbar_D09G010030.1	Gbar_D09G010030.1	GbHDT1D.1	0.009010245	0.06298406	0.14305596	2.099468568	Purifying selection	Segmental
GbHDT3A.2	Gbar_D11G036570.1	Gbar_D11G036570.1	GbHDT3D.1	0.018447187	0.04825246	0.382305665	1.608415218	Purifying selection	Segmental
GhHDT3A.1	Ghir_D01G021720.1	Ghir_D01G021720.1	GhHDT3D.1	0.042065664	0.07531594	0.558522702	2.510531459	Purifying selection	Segmental
GhHDT1A.1	Ghir_D05G008730.1	Ghir_D05G008730.1	GhHDT1D.1	0.007526405	0.03963186	0.189907947	1.321062034	Purifying selection	Segmental
GhHDT1A.2	Ghir_D09G009840.1	Ghir_D09G009840.1	GhHDT1D.2	0.007681542	0.06383745	0.120329713	2.127914992	Purifying selection	Segmental
GhHDT3A.2	Ghir_D11G035640.1	Ghir_D11G035640.1	GhHDT3D.2	0.018451552	0.04269717	0.432149327	1.423238868	Purifying selection	Segmental

The ratio of non-synonymous to synonymous substitutions (Ka/Ks) provides an important measure of the pressure of evolutionary assortment on the relevant amino acids (Hurst, 2002). This ratio was measured for the duplicated *Gossypium* HD2 gene pairs (paralogous). The result of this calculation showed that all the duplicated HD2s had a Ka/Ks ratio < 1 (**Table 4**). Therefore, duplicated gene pairs in cotton HD2 members were under powerful purifying selection pressure.

**Figure 8:**
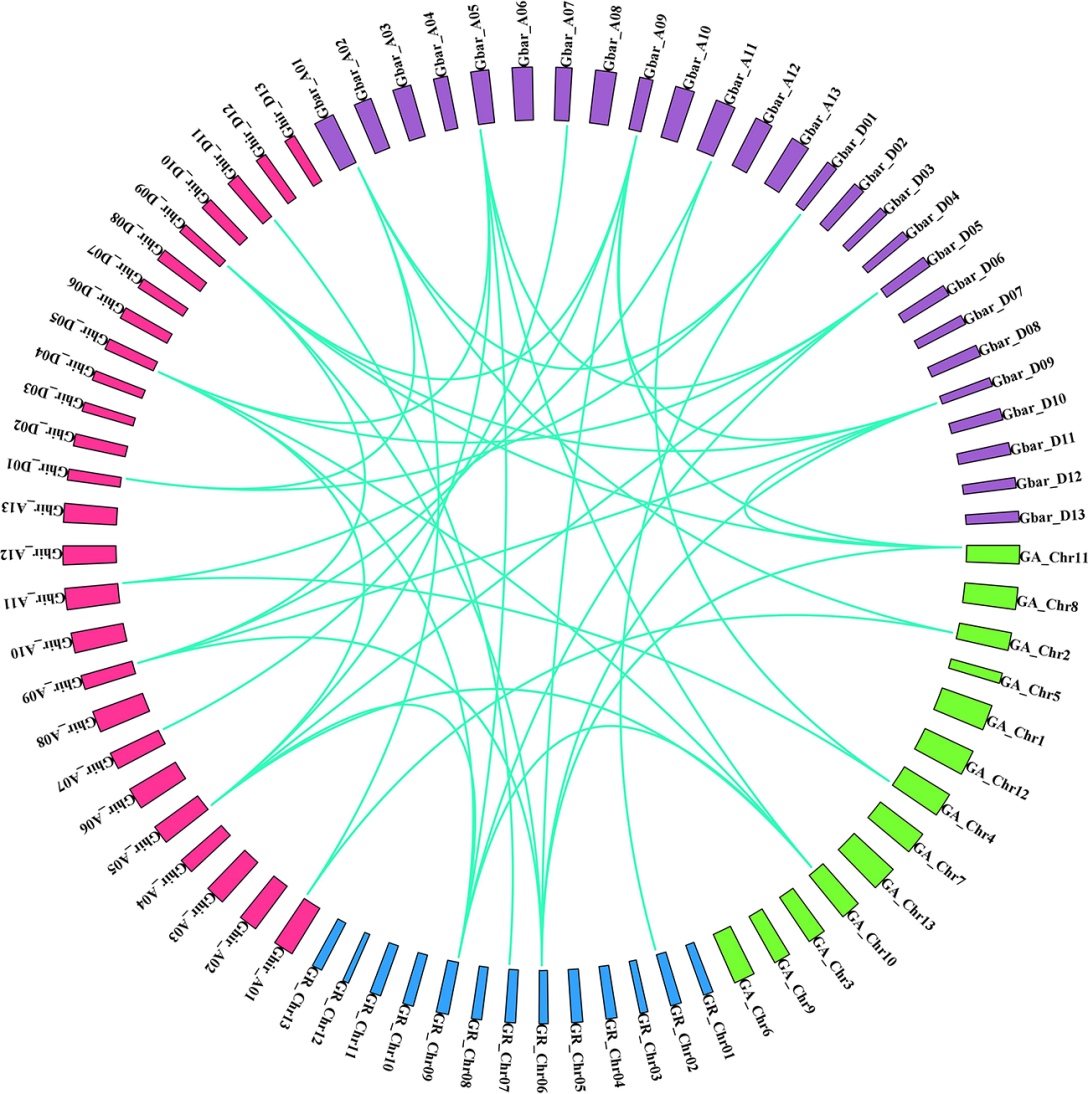
Syntenic associations in orthologous HD2 gene pairs in four *Gossypium* species (green: *G. arborium*; blue: *G. raimondii*; pink: *G. hirsutum*; violet: *G. barbadense*).

A pair of homologous genes that have diverged in dissimilar species during a speciation event is known as an ortholog. The orthologous associations among the HD2 members in the four cotton species were determined. Specifically, orthologous genes among the HD2 members were identified *via* sequence similarity. Orthologs having sequence similarity equal to or greater than 90% in both cDNA and amino acid (aa) composition were selected for further evolutionary study. The selection pressure and probable functional divergence of *Gossypium* HD2s genes were examined through the calculation of Ka, Ks, and Ka/Ks ratio among the orthologs (A vs. D, A_T_ vs. A, D_T_ vs. D, A_T_ vs. A_T_, and D_T_ vs. D_T_) and within the homeologs (A_T_ vs. D_T_). Notably, the Ka values of *Gossypium* HDT3 orthologs (group IV and V HD2s; GaHDT3.2/GrHDT3.2, GhHDT3A.1/GhHDT3D.1, GbHDT3D.1/GrHDT3.2, GhHDT3A.2/GbHDT3A.2, and GhHDT3D.2/GbHDT3D.1) and HDT1 orthologs (GbHDT1A.1/GaHDT1.2) were higher than those of other HD2 gene pairs, suggesting that these ortholog pairs underwent more rapid evolution of the relevant genes. With a Ka/Ks ratio <1, this analysis revealed that negative selection had been exerted on HD2 orthologous genes (**Supplementary Table 2**), and some orthologous gene pairs had experienced directional selection. The Ka/Ks ratio was higher for HDT3 orthologous gene pairs, in A vs D, A_T_ vs D_T_, A_T_ vs A, D_T_ vs D, A_T_ vs A_T_, and D_T_ vs D_T_. There were three HDT3 orthologous gene pairs (GaHDT3.2/GrHDT3.2, GbHDT3A.1/GaHDT3.1, and GhHDT3D.2/GrHDT3.2) that had a Ka/Ks ratio > 1, meaning that they might have undergone adaptation to certain advantageous alleles that might play an important role in cotton. These outcomes suggest that the diploid cotton HDT3 member was under greater evolutionary pressure, and that the evolution of the A genome might have been faster than that of the D sub-genome (**Figure 8** and **Supplementary Table 2**).

### *Cis-* regulatory elements in the promoter region of GhHD2s

**Figure 9:**
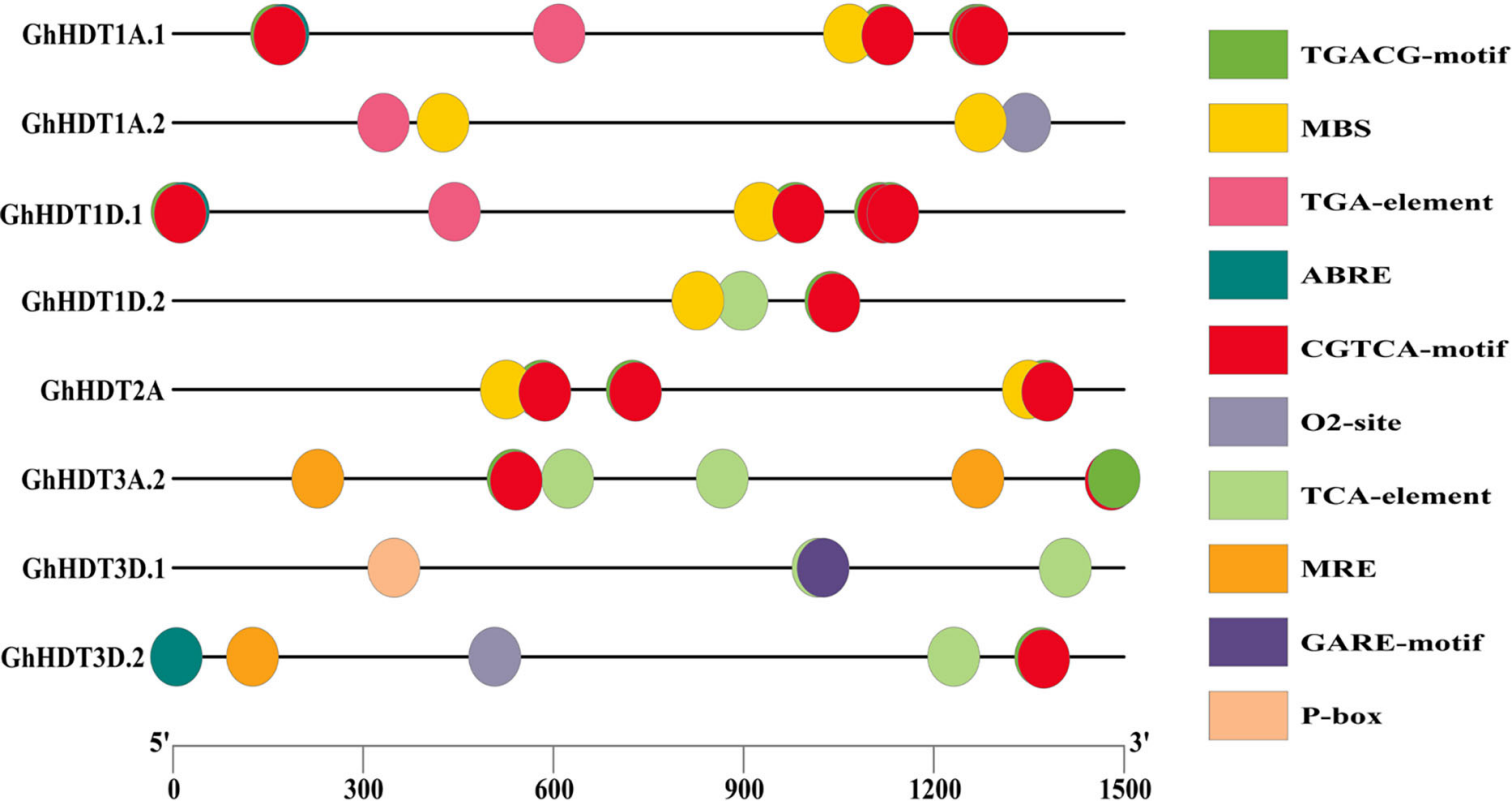
Results of investigation of the *cis*-regulatory elements of GhHD2 members. Micro-parts shown in distinct colors represent the sequence of the putative elements.

Promoters are regions of DNA that drive the initiation of transcription of a certain gene; these promoters are situated near the transcription start site (TSS) of the corresponding gene. In the present study, we searched for *cis*-regulatory elements in the 1.5 kb upstream promoter region of the identified GhHD2s. The outcomes of this analysis are listed in **Supplementary Tables 3A, B**. The results revealed that *cis*-regulatory elements like ABRE were most abundant, followed by TGACG and CGTCA motifs. Additionally, TCA-element was abundant in the promoter regions of identified GhHD2s (**Figure 9**). These outcomes indicate that GhHD2 genes are associated with plant resistance to environmental stress conditions, including drought and salt stress.

It is interesting that the GhHD2 genes, namely GhHDT1A.1, GhHDT1A.2, GhHDT1D.1, GhHDT1D.2, GhHDT2A, GhHDT3A.2, GhHDT3D.1, and GhHDT3D.2, were found to comprise several hormone-responsive *cis*-elements, such as TCA-element (salicylic acid responsiveness), TGA-element (auxin-responsive element), ABRE (abscisic acid responsiveness), CGTCA-motif (MeJA-responsiveness), P-box (gibberellin-responsive), TGACG-motif (MeJA-responsiveness), and GARE-motif (gibberellin-responsive), in their promoter region (**Figure 9**). MeJA is reported to stimulate the production of defensive compounds that may be used against pathogens, drought stress, heavy metal stress, low temperatures, and salt stress (Yu et al., 2019), while auxin plays a crucial role in plant responses to adverse biotic and abiotic conditions (Sharma et al., 2015). In addition to pathogenesis-related resistance, drought and heat stress also trigger the production of salicylic acid (SA), and this also reduces the concentrations of Na+ and Clˉ ions caused by salt stress conditions (Yuan and Lin, 2008; Emamverdian et al., 2020). Plant growth hormones, such as gibberellins (GAs) are important in increasing resistance under abiotic stresses (Emamverdian et al., 2020), and plants respond to downstream stress by integrating various stress signals *via* the abscisic acid (ABA) hormone (Tuteja, 2007). Overall, these promoter analyses show that these genes play an important role in providing tolerance to drought and salt stress conditions.

### HD2 expression profiles under salt and drought stress

**Figure 10:**
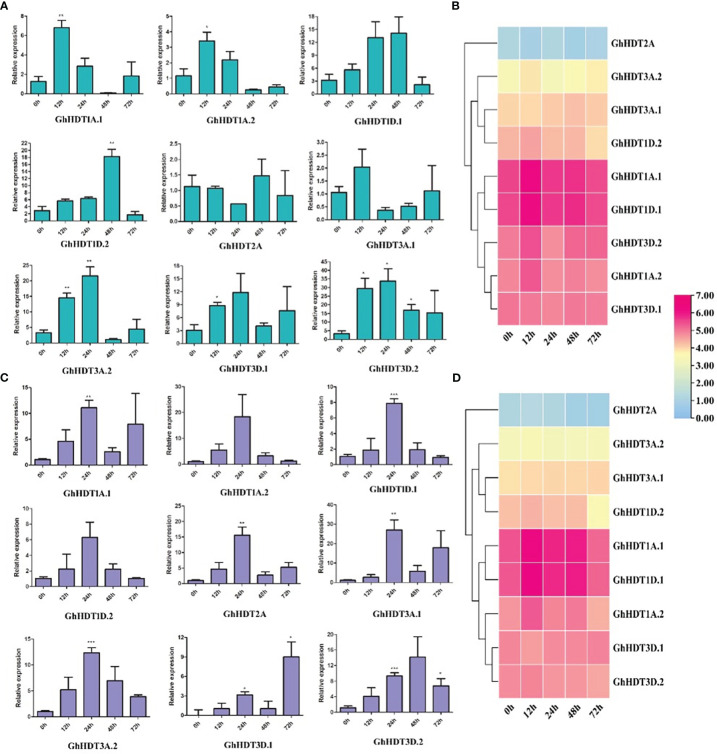
The qRT-PCR expression profiles of putative HD2 genes in *G. hirsutum*. Bars represent expression levels at 12, 24, 48, and 72 h of **(A)** drought (20% PEG solutions PEG8000) and **(C)** salt (300 MM) stress treatment, as compared to control levels at 0 h. Ubiquitin was taken as a normalizing control. Three replicates were used for each experiment. Two-tailed Student’s t-tests were used for statistical analysis; data are plotted in the form mean ± s.d, with error bars representing standard deviations. Significant differences are denoted with asterisks: *P < 0 0.1; **P < 0 0.01; ***P < 0 0.001. Right panels show expression profiles of HD2 genes under **(B)** drought and **(D)** salt conditions according to RNA-Seq data, using log2FPKM values.

In order to determine the expression pattern of HD2 genes under drought and salt stress, HD2 genes were analyzed *via* the leaf RNA-Seq data under both types of stress. Nine identified GhHD2 genes showed differential expression, with three genes (GhHDT1A.2, GhHDT3D.1, and GhHDT3D.2) exhibiting higher expression during drought (**Figure 10B**) and salt stress (**Figure 10D**) compared to a control. Furthermore, these nine putative genes (GhHDT2A, GhHDT3A.1, GhHDT3A.2, GhHDT1D.2, GhHDT1A.1, GhHDT1A.2, GhHDT1D.1, GhHDT3D.1, and GhHDT3D.2) were validated through qRT-PCR. The list of primers is provided in **Table 5**. In dought, the majority of GhHD2s exhibited stronger responses at 12h (**Figure 10A**), with the exception of GhHDT2A, while the strongest response was that of GhHDT3D.2 with respect to fold change (FC) compared with the control at 12 h (FC >29), 24h (FC >33), 48h (>16), and 72h (>15) (**Figure 10A**). Under salt stress conditions, the entire set of GhHD2 members exhibited higher levels of expression at 24h compared with the control (**Figure 10C**), while GhHDT3D.2 exhibited the highest level of expression in comparison to the control at 12 h (FC >4), 24h (FC >9), 48h (>14), and 72h (> 6). Overall, this analysis shows that these HD2 members might play a crucial role in regulating responses to drought and salt stress conditions.

**Table 5:** List of primers (forward and reverse) used in qRT-PCR validation.

Drought	Forward primer (5’ to 3’)	Reverse primer (5’ to 3’)
**GhHDT3A.1**	TCTCCTTTCGCTTTGGTGTTG	GGGCAACCTGTGAAAGATGC
**GhHDT1A.1**	CCCAGGCTTCACTTGGAGAG	ATTGAGGGCAACTCTGGTGG
**GhHDT2A**	CCACCAGAGTTGCCCTCAAT	GCATTAGCCTTGGCGAGGAA
**GhHDT1A.2**	ACTTTTGTCCCAGAGGAAGGC	TCCCAGCATTAGCCGTTTTTG
**GhHDT3A.2**	TACGCCTCAAGGTTCTGAGT	CGTGGTAGGATCAGATGCTGT
**GhHDT3D.1**	CAAAGCTGAAAGCGGGTGGA	CCACAGGACTTGCAAGGGAA
**GhHDT1D.1**	AAGAAGGGTGGACACACAGC	CAGACCCGAACGACTTTGGA
**GhHDT1D.2**	CAGCGACACCTCAGAAGACA	TCGGAACCAAAGGACTTGCT
**GhHDT3D.2**	ACGCCTCAAGGTAGTGGTTC	ACCTGTTTTGACGTGGTAGGA
Salt		
**GhHDT3A.1**	TCTCCTTTCGCTTTGGTGTTG	GGGCAACCTGTGAAAGATGC
**GhHDT1A.1**	AAGAAGGGTGGACACACAGC	ACCACAAGAGAATTGACCACCA
**GhHDT2A**	AAGGGTGGACACACCACATC	CTCTAAACCGCCCTCAGACC
**GhHDT1A.2**	GTCCCAGAGGAAGGCTCTGAT	GGTTTCCCAGCATTAGCCGTT
**GhHDT3A.2**	TACGCCTCAAGGTTCTGAGT	CGTGGTAGGATCAGATGCTGT
**GhHDT3D.1**	GAAGGCAAAGCTGAAAGCGG	CACCGGACTTTGGAGTCTGC
**GhHDT1D.1**	AAGAAGGGTGGACACACAGC	CAGACCCGAACGACTTTGGA
**GhHDT1D.2**	CAGCGACACCTCAGAAGACA	TCGGAACCAAAGGACTTGCT
**GhHDT3D.2**	ACGCCTCAAGGTAGTGGTTC	ACCTGTTTTGACGTGGTAGGA

### Co-expression network, pathways, and gene ontology analysis of GhHDT3D.2, a gene strongly expressed under drought and salt stress conditions at different time scales

The GhHDT3D.2 gene was selected for analysis of the co-expression network, pathways, and gene ontology due to its higher levels of expression under both types of stress. Co-expressed genes with GhHDT3D.2 were explored using log2FPKM expression values. A total of 15 genes positively and 14 negatively co-expressed with GhHDT3D.2 (PCoEGs and NCoEGs) were identified under drought stress conditions (**Supplementary Figures 1A, B**; **Supplementary Table 4**), while 80 such PCoEGs and 237 such NCoEGs (**Supplementary Figures 1C, D**) were identified under salt stress conditions (**Supplementary Table 4**).

In order to understand the functional pathways and molecular importance of the PCoEGs and NCoEGs of GhHDT3D.2, a PageMan pathway analysis was performed. For drought stress conditions, this analysis demonstrated that there was higher expression of fatty acid metabolism and auxin response factors (ARFs) in relation to NCoEGs of GhHDT3D.2 and vacuolar-sorting protein (SNF7) in relation to PCoEGs of GhHDT3D.2 (**Supplementary Figure 1E**). Moreover, for salt stress conditions, diacylglycerol kinase (**Supplementary Figure 1F**) exhibited higher expression at all time intervals, while the highest level of expression among the PCoEGs of GhHDT3D.2 at 12h was that of brassinosteroid hormone metabolism; among the NCoEGs of GhHDT3D.2, the highest level of expression was that of phenypropanoid and flavonoids (**Supplementary Figure 1G**). Transcription factors such as basic leucine zipper (bZIP), WRKY, plant homeo-domain (PHD), and heat shock factors (HSF) exhibited expression with PCoEGs of GhHDT3D.2, and abiotic stress-responsive genes exhibited expression with NCoEGs of GhHDT3D.2 under salt stress conditions (**Supplementary Figure 1H**).

A gene ontology (GO) analysis was carried out to examine all the identified co-expressed genes and identify their functional roles. GO divided into three categories: biological processes (BPs), cellular components (CCs), and molecular functions (MFs). The significant PCoEGs and NCoEGs of GhHDT3D.2 were selected for gene ontology analysis. Under drought stress, enriched MFs were hydrolase activity, carbohydrate derivation binding, and purine nucleotide binding; enriched BPs were establishment and localization; and enriched CCs were the membrane, endomembrane system, and cytoplasmic part (**Supplementary Figure 1I** and **Supplementary Table 5**). Likewise, under salt stress conditions, enriched BPs were response to water deprivation, abiotic stimulus, salt stress, ethylene, the auxin-activated signaling pathway, and gibberellins (**Supplementary Figure 1J** and **Supplementary Table 5**); enriched MFs were transcription factor activity, transporter activity, calmodulin binding, phosphor-transferase activity, and hydrolase activity; and enriched CCs were the vacuolar membrane, golgi apparatus, plasma membrane, cytoskeletal part, and cell part (**Supplementary Figure 1J** and **Supplementary Table 5**).

## Discussion

Epigenetics is primarily concerned with changes in heritable gene expression without the involvement of variations in DNA sequences. Such changes are essential to the development and growth of plants in response to several environmental stimuli, and modification of histone is closely associated with the control of gene expression. HDACs are also called lysine deacetylases; they regulate gene expression, with core histones (H2A, H2B, H3, and H4) and acetyl groups being removed by HDACs to repress their transcription (Makarevitch et al., 2015). Earlier studies have demonstrated that HDACs are crucial in enabling plants to respond to a variety of environmental stresses (Liu et al., 2014), and for growth, development (Ma et al., 2013), and genome stability (Luo et al., 2012). There are three members of the set of HDACs, identified as RPD3/HDA1, SIR2, and HD2, in which HD2 proteins have been found to differ from those of the other two types of histone deacetylases.

In the present study, four, nine, nine, and five HD2 members were identified in the four sequenced species of Gossypium (*G. arboreum*, *G. hirsutum*, *G. barbadense*, and *G. raimondii*, respectively) (**Table 1**). The larger numbers of HD2 members in *G. hirsutum* and *G. barbadense* might be due to their larger genomes (~2.30 Gb and ~2.22 Gb, respectively; Hu et al., 2019) as compared to *G. arborum* (1,746 Mb) and *G. raimondii* (885 Mb) (Li et al., 2014). All cotton HD2 members are located in the nucleus, and many earlier reports have also revealed that HD2 members are nucleus-localized (Zhou et al., 2004; Sridha and Wu, 2006), while only a few members are present in the nucleolus (Lusser et al., 1997; Earley et al., 2006).

As per the domain analysis (**Figure 1**), all conserved cotton HD2 members contained the HD2 label, a deacetyl/catalytic domain, phosphorylation sites, mono- and bipartite NLS motifs, and a zinc finger domain from N-terminus to C-terminus. In contrast, GrHDT3.1, 4, GhHDT2A, and GbHDT2A did not contain a bipartite NLS motif or zinc finger domain, and GhHDT1D.1 did not contain a zinc finger at the C-terminus end (**Figure 2**). It has been previously reported that *Arabidopsis thaliana* and *Medicago truncatula* are also devoid of zinc finger at their C-terminal end (Bourque et al., 2016). On the basis of the presence or absence of Zn^2+^ finger at the C-terminus, the HD2 family can be divided into two groups: the group with a conserved Zn^2+^- finger domain at the C-terminal end of HD2 is referred to as the Gr1 group, and the group of HD2s that lack a conserved Zn^2+^- finger domain at the C-terminal end is the Gr2 group (Bourque et al., 2016). This result indicates that Gr2 HD2 genes might contribute to the expansion of cotton HD2s. Overall, cotton HD2s have a similar domain organization to that reported previously (Demetriou et al., 2009).

HD2s are a part of a small gene family (Grandperret et al., 2014) whose members number between one (in longan and potato) and four (in *Arabidopsis* and maize). The N-terminal domain of the protein sequence includes conserved octapeptide MEFWGVEV; previously, the MEFWG sequence has been reported to be an explicit structural feature of the HD2 gene family (Aravind and Koonin, 1998), and these starting five residues might play a significant role in gene regulation. The catalytic domain of the HD2 gene family is made up of 100 amino acid residues and contains the conserved regions (Bourque et al., 2016). These residues comprise two highly conserved acidic amino acids that have histidine residue at position 25, surrounded by the hydrophobic amino acids, and aspartic acid residue at position 69. The catalytic activity of HD2s depends on these two residues, which represent conserved motifs (Aravind and Koonin, 1998; Dangl et al., 2001; Ding et al., 2012). Residues such as leucine, present at position 26, are also highly conserved and play an important role in catalytic machinery or ligand binding (Bourque et al., 2016) (**Figure 3**). A central large acidic domain is highly variable in length as well as in sequence, also known as the regulatory domain of HD2s. Aspartic acid residue detected in the entire HD2 domain comprises Ser and/or Thr residues as phosphorylation sites for mitogen-activated protein kinases (MAPKs) and casein kinase 2α (CK2α) (Grandperret et al., 2014; Bourque et al., 2016) (**Figure 3**). CK2α is located in the nucleus and regulates many biological processes through phosphorylation of numerous distinct proteins. All HD2s comprise a conserved and well-characterized monopartite NLS domain along with the bipartite NLS sequence KK(K/R) that is found in ten to twelve residues before the monopartite NLS motif (Bourque et al., 2016).

The putative N-terminal HD2 label that are MEFWG sequence (well-conserved motif) amino acid regions require to control the gene expression. To see the domain conservation, cotton HD2 members were aligned with *A. thaliana* HD2s (**Figure 3** and **Supplementary Table 1**). The outcomes of the motif analyses suggested that the amino acids at these conserved regions of cotton HD2s exhibit a very high degree of homology with AtHD2s. All the cotton HD2s have a conserved pentapeptide HD2 label at the N-terminus region and conserved deacetylase sites, as these are present in all AtHD2s. Subsequently, the zinc finger properties of cotton HD2s were examined; GbHD2A, GhHD2A, GrHD3.1, and GrHD4 were found to lack zinc fingers at their C-terminus region, indicating that these are more similar to HDT2 and HDT4 of *Arabidopsis*, each of which is devoid of zinc finger (**Figure 3**).

The phylogenetic tree indicated the arrangement of the cotton HD2 members. The set of HD2 genes in cotton seems to contain numerous members that have orthologs as distant as the common ancestor of *Arabidopsis, T. cacao*, and in some cases other plant species (Bourque et al., 2016). Previous reports have indicated that Gr2 HD2s evolved only in angiosperms. The absence of zinc fingers only in angiosperm indicates that Gr1 HD2s represent the ancestral form of the gene (Bourque et al., 2016). Group IIIa and X members, which contain mostly Gr2 HD2s, were thus the latest group to evolve. HD2s from lycophytes were found to cluster into Groups Ia and VIIIb; bryophytes clustered into Group VIIc; and lower plants fell into Groups IV, VIIIb, and X; none of the cotton HD2 members belonged to Group IV, VIIIb, or VIIc. Basal angiosperms clustered into Group Ia, while eleven cotton HD2 genes belonged to this group (**Figure 4**). Furthermore, only higher plants were represented in Group V, VI, and X and in five subgroups (Ia, IIa, IIb, IIIa, IIIb, VIIa, VIIb,VIIIa) of Groups I, II, III, VII, and VIII, while HD2s were found to exist in IIa, IIb, V, VIIb, and VIIIa, which were entirely monocot-specific (**Figure 4**). With the exception of Group VIIc, HD2 members were present in both monocot and eudicot plants. The evolutionary paths of all these groups’ evolutionary paths diverged before monocots and dicots, with a common ancestor.

The motif arrangements and gene patterns of cotton HD2 members were conserved in most of the groups, with some exceptions (**Figure 5**), suggesting their functional conservation. GaHDT3.2 comprised a smaller number of introns (5), while GbHDT2A contained the largest number (11) across all cotton HD2 genes. As per previous reports, more advanced species contained smaller numbers of introns in their genomes (Roy and Gilbert, 2005), while an increased number of introns results in new functions (Qanmber et al., 2019).

The duplication of genes plays a crucial role in the functional divergence of genes (Takata and Taniguchi, 2015). Potential gene duplication events in the cotton HD2 gene family were evaluated to assess the probable relationships among members of the HD2 group (**Table 4**). The results with regard to gene duplication events among *Gossypium* species suggested the remarkable hypothesis that recent duplication has taken place in cotton HD2s after the divergence of *G. riamondii* and *G. arboreum* and led to the formation of *G. barbadense* and *G. hirsutum* (Li et al., 2014). In the present study, segmental duplication (as opposed to tandem duplication) was found to play a dominant role in the enlargement of *Gossypium* HD2 members (**Figure 7**), as these gene pairs were present in different chromosomes. Positive (directional or Darwinian) selection encourages the extent of advantageous alleles, and negative or purifying selection hampers the proliferation of deleterious alleles (Altenhoff and Dessimoz, 2009). In this study, a Ka/Ks ratio < 1 was observed for all paralogous HD2 gene pairs in cotton, implying strong purifying selection pressure, meaning that this contributes to maintaining their function in the *Gossypium* HD2 gene. Exploration of these duplicated genes showed that the functional role of HD2 genes in cotton has not greatly diverged in the course of subsequent evolution. However, some of the orthologous gene pairs in *Gossypium* exhibited signs of directional selection (**Figure 8** and **Supplementary Table 2**), which plays a crucial role in the expansion of beneficial alleles that might be playing important role in cotton. These results support further functional analysis of this gene family.

Analysis of expression profiles in *G. hirsutum via* qRT-PCR revealed higher levels of expression of GhHDT3D.2 at all time intervals under drought stress conditions (**Figure 10A**). Under salt stress, higher levels of expression of GhHDT1A.1, GhHDT2A, GhHDT3A.1, GhHDT3A.2, GhHDT3D.1, and GhHDT3D.2 were observed at all time intervals, with the exception of GhHDT1A.2, GhHDT1D.1, and GhHDT1D.2 (**Figure 10B**). GhHDT3D.2 was the common gene that exhibited significantly higher levels of expression under both types of stress. These outcomes of this analysis suggest that the GhHDT3D.2 gene might play a crucial role in providing resistance against drought and salt stress, and would probably be a suitable target to protect the cotton plant from both these abiotic stresses.

To further elucidate the function of the GhHDT3D.2 gene, the co-expression network of this gene was studied (**Supplementary Table 4**). This analysis showed the presence of SNF7 transcription factor (TF) in PCoEGs of GhHDT3D.2, whose transcript accumulates during drought stress in *Populus davidiana* (Mun et al., 2017). PCoEGs of GhHDT3D.2 under drought conditions also comprised zinc finger-RING-types, a tify domain, and a GTP binding domain. Zinc-finger proteins play a crucial role in responses to abiotic stimuli, such as drought, extreme temperatures, reactive oxygen species, salt, and toxic metals, in plants. They mostly operate as E3 ubiquitin ligases and contain a conserved RING domain (Han et al., 2022); tify and GTP binding domain functions have also been identified in drought stress responses in cotton and Chinese cabbage (Zhao et al., 2016; Chen et al., 2021) (**Supplementary Figure 1A** and **Supplementary Table 4**). NCoEGs of GhHDT3D.2 contained a pentatricopeptide repeat, which plays an important role in drought, salt, and cold stress responses in *Arabidopsis* (Jiang et al., 2015) (**Supplementary Figure 1B** and **Supplementary Table 4**). Finally, SNF7 transcription factor and auxin response factor (ARF) were identified in the PageMan pathways of PCoEGs of GhHDT3D.2 (**Supplementary Figure 1E**); these play a crucial role in providing tolerance to drought stress conditions.

Moreover, PCoEGs of GhHDT3D.2 under salt stress comprised WD40 repeats, a WRKY domain, glycoside hydrolase, a thioredoxin domain, and armadillo-like and leucine-rich repeats (**Supplementary Figure 1C**). O f these PCoEGs, WD40 protein is regulated *via* salt stress in rice, and suggests a crucial role in providing tolerance to salt stress (Huang et al., 2008). One of the largest TF families, the WRKY family is known to participate in a number of abiotic stress responses (Xu et al., 2018). The expression of thioredoxin improves salt tolerance in *Brassica napus* (Ji et al., 2020); additionally, in *Solanum lycopersicum*, glycoside hydrolase functions as a putative biomarker for salt stress tolerance (Reyes-Perez et al., 2019). PageMan pathways analysis of the PCoEGs of GhHDT3D.2 showed higher levels of expression of phospholipid synthesis, which plays a regulatory role in the salt stress response (Han and Yang, 2021) (**Supplementary Figure 1F**), while metabolism of brassinosteroid hormones reduces the negative consequences of salt stress (Su et al., 2020) (**Supplementary Figure 1G**). Furthermore, the NCoEGs of GhHDT3D.2 under salt stress contained GRAS TF, lateral organ boundaries, and tubby-like protein. In transgenic *Arabidopsis*, over-expression of GRAS improves salt stress tolerance and plant growth (Zhang et al., 2020). The expression of lateral organ boundaries (LOB) has been found to be upregulated in *Vitis vinifera* under salt stress *via* treatment with ABA (Grimplet et al., 2017), while tubby-like protein transcription factor (TLP TF) has been reported to play an important role in increasing tolerance to salt stress (Bano et al., 2021) (**Supplementary Figure 1D**). PageMan pathways analysis of the NCoEGs of GhHDT3D.2 revealed the presence of phenylpropanoid, resulting in elevated salt stress tolerance (Sharma et al., 2019). Additionally, the flavonoid pathway secondary metabolism enhances salt stress tolerance by scavenging free radicals (Jan et al., 2021) (**Supplementary Figure 1G**). NCoEGs of GhHDT3D.2 also exhibited higher expression during abiotic drought and salt stress conditions, as did GATA transcription factor (**Supplementary Figure 1H**), which exhibits higher levels of expression in rice under salt stress (Zhang et al., 2021).

In terms of the gene ontology functions of significant PCoEGs and NCoEGs of GhHDT3D.2 under drought conditions, enriched molecular functions were hydrolase activity and carbohydrate derivation binding (**Supplementary Figure 1I**). In response to drought stress, hydrolase acts as a negative regulator in peach (He et al., 2022). By participating in NADPH oxidase-mediated ROS generation, carbohydrate-binding protein mediates drought-stress tolerance in rice (Jing et al., 2022). The biological processes involved in salt stress are enhanced by responses to water deprivation, abiotic stimulation, salt stress, ethylene, auxin-activated signaling, and gibberellin pathways (**Supplementary Figure 1J**), in which ethylene modulates salinity stress responses primarily by maintaining Na+/K+ homeostasis, reactive oxygen species (ROS), and nutrients, through stimulation of antioxidant defense (Riyazuddin et al., 2020). Additionally, enriched molecular functions were transporter activity, calmodulin binding, and phosphotransferase activity (**Supplementary Figure 1J**); in this regard, it has been reported that ion transporters are crucial for salt stress tolerance and help in the breeding of crop cultivars with high salt tolerance (Huang et al., 2020). Additionally, Ca^2+^ sensor calmodulin1 (CaM1) negatively regulates salt stress tolerance through interaction with calmodulin binding transcription activator 4 (CAMTA4) in *Hordeum vulgare* (Shen et al., 2020). Overall, our research demonstrates the significance of the PCoEGs and NCoEGs of GhHDT3D.2 under salt and drought conditions. Thus, the GhHDT3D.2 gene can be used to improve the performance of cotton under drought and salt stress conditions.

## Conclusion

The cotton HD2 gene family was comprehensively analyzed in the present study. A total of 27 cotton HD2 proteins with a conserved HD2 label, deacetyl/catalytic domain, phosphorylation sites, mono- and bipartite NLS motifs, and a zinc finger domain from N-terminus to C-terminus were identified in four cotton genomes (G*. arboreum, G. raimondii*, G. *barbadense*, and *G. hirsutum*). These proteins showed noticeable similarities in terms of their common conserved motifs, protein domains, and gene structures, with related functions. A total of ten pairs of duplicated genes were identified; these occurred in paralogous gene pairs in four cotton species, and all had been underwent purifying selection pressure. The expression profiles of GhHD2s showed significantly higher levels of expression of GhHDT3D.2 underwent drought and salt stress conditions at all time intervals (0, 12, 24, 48, and 72h). Finally, the PCoEGs and NCoEGs of GhHDT3D.2 revealed the important functions and pathways that play a crucial role in both of these stress conditions. The present study strengthens the field’s understanding of HD2 genes in cotton at the levels of structure, functions, pathways, evolution, and expression. This study offers an improved understanding of the biological involvement of cotton HD2 members, and this will provide benefits in the form of improved cotton production in the presence of drought and salt stress conditions.

## Data availability statement

The datasets presented in this study can be found in online repositories. The names of the repository/repositories and accession number(s) can be found in the article/**Supplementary Material**.

## Author contributions

NB carried out the bioinformatics analysis, designed the study, and drafted the manuscript. SF and RL performed qRT-PCR validation. CM and SB participated in supervision of the study. All authors contributed to the article and approved the submitted version.

## Supplementary material

Supplementary Figure 1 Co-expression network, pathways, and gene ontology analysis of genes co-expressed with GhHDT3D.2. **(A, B)** Positively and negatively co-expressed genes (PCoEGs and NCoEGs) of GhHDT3D.2 in drought conditions and **(C, D)** in salt conditions at different time intervals. Ovals (nodes) represent transcripts, and lines (edges) depict transcriptional interactions between GhHD2s and transcripts. **(E)** PageMan-based pathway classification of positively and negatively co-expressed genes (PCoEGs and NCoEGs) under drought conditions and **(F–H)** under salt stress conditions at 0, 12, 24, 48, and 72h. Green and red bin colors represent log2 expression values provided in the scale bar. **(I)** Gene ontology classification under drought and **(J)** salt stress conditions.
