# Supplementary figures and images for: Genome-wide identification and expression analysis of the HD2 protein family and its response to drought and salt stress in Gossypium species
Source: Front Plant Sci. 2023 Feb 13;14:1109031. doi: 10.3389/fpls.2023.1109031 (PMC9968887; doi:10.3389/fpls.2023.1109031)

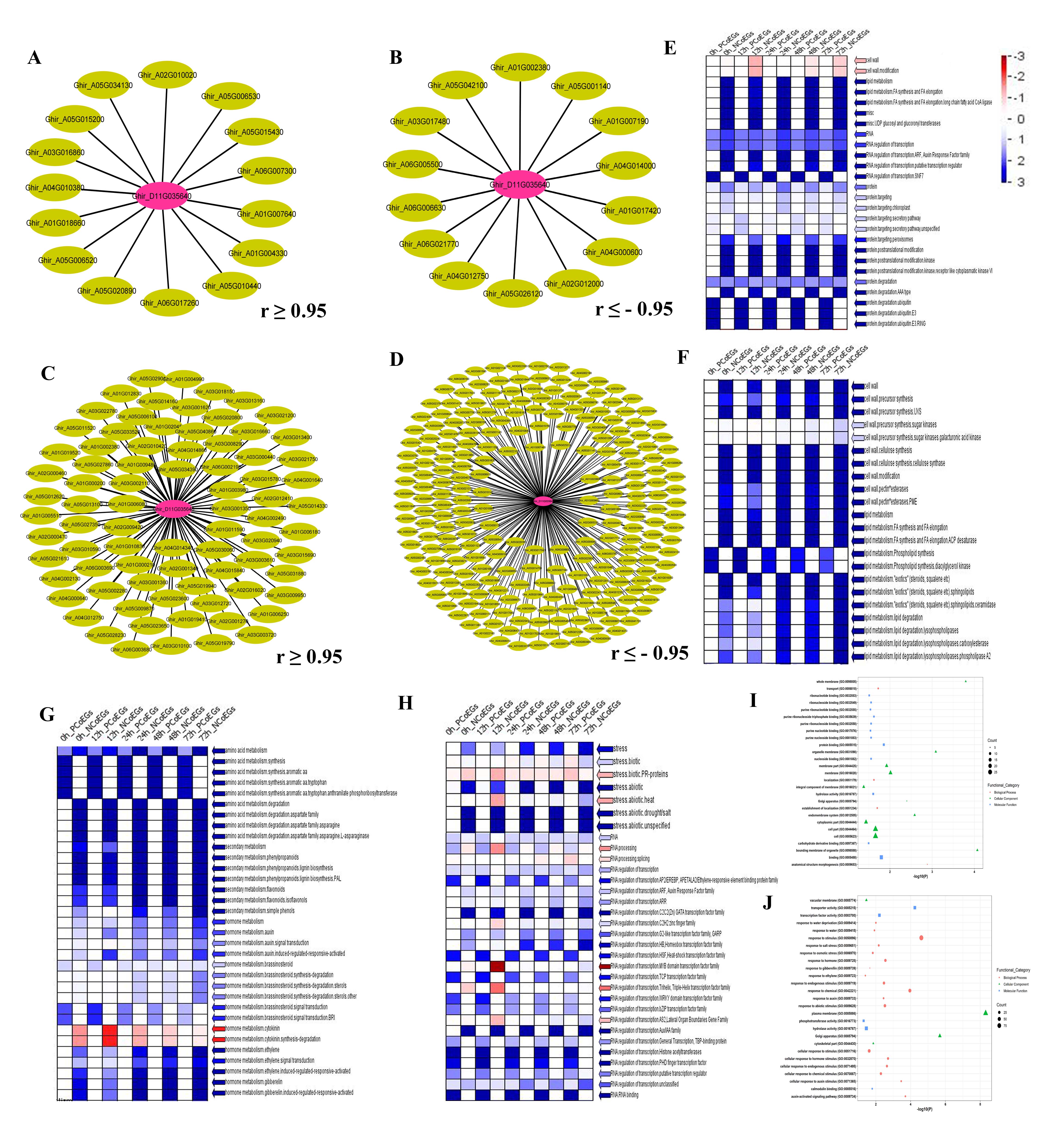

Supplement: Supplementary Figure 1 — Co-expression network, pathways, and gene ontology analysis of genes co-expressed with GhHDT3D.2. (A, B) Positively and negatively co-expressed genes (PCoEGs and NCoEGs) of GhHDT3D.2 in drought conditions and (C, D) in salt conditions at different time intervals. Ovals (nodes) represent transcripts, and lines (edges) depict transcriptional interactions between GhHD2s and transcripts. (E) PageMan-based pathway classification of positively and negatively co-expressed genes (PCoEGs and NCoEGs) under drought conditions and (F–H) under salt stress conditions at 0, 12, 24, 48, and 72h. Green and red bin colors represent log2 expression values provided in the scale bar. (I) Gene ontology classification under drought and (J) salt stress conditions. [file Image_1.jpeg]
